# Association of the genetic variants (‐794 CATT5‐8 and ‐173 G > C) of macrophage migration inhibitory factor (MIF) with higher soluble levels of MIF and TNFα in women with breast cancer

**DOI:** 10.1002/jcla.23209

**Published:** 2020-01-24

**Authors:** Guadalupe Avalos‐Navarro, Alicia Del Toro‐Arreola, Adrián Daneri‐Navarro, Antonio Quintero‐Ramos, Luis Alberto Bautista‐Herrera, Ramon Antonio Franco Topete, Brian Uriel Anaya Macias, David Israel Javalera Castro, Andrés de Jesús Morán‐Mendoza, Antonio Oceguera‐Villanueva, Antonio Topete‐Camacho, José Francisco Muñoz‐Valle

**Affiliations:** ^1^ Laboratorio de Inmunología Departamento de Fisiología CUCS Universidad de Guadalajara Guadalajara México; ^2^ Departamento de Biología Molecular y Genómica Instituto de Investigación en Ciencias Biomédicas (IICB) Universidad de Guadalajara Guadalajara México; ^3^ Laboratorio de Patología Departamento de Patología y Microbiología CUCS Universidad de Guadalajara Guadalajara México; ^4^ OPD Hospital Civil de Guadalajara “Nuevo Hospital Civil, Juan I. Menchaca” Guadalajara México; ^5^ Hospital de Especialidades Centro Médico Nacional de Occidente IMSS Guadalajara Mexico; ^6^ Instituto Jalisciense de Cancerología Secretaría de Salud Guadalajara Mexico

**Keywords:** breast cancer, migration inhibitory factor, polymorphism, soluble levels, TNFa

## Abstract

**Background:**

Functional variants ‐173 G > C (rs755622) and ‐794CATT_5‐8_ (rs5844572) *MIF* gene have been associated with the risk in several types of cancer, as well as with the increase of soluble levels of MIF and TNFα. However, in previous studies contradictory and uncertain results have been presented on the implication of *MIF* polymorphisms with the association in cancer, specifically in breast cancer (BC). We investigated whether the variants are associated with the susceptibility to develop BC and the soluble levels of MIF and TNFα in women with BC from western Mexico.

**Materials and methods:**

A total of 152 women with BC and 182 control subjects (CS) were enrolled in this study. The determination of genotypes ‐173 G > C and ‐794 CATT_5‐8_ *MIF* polymorphisms was performed by PCR‐RFLP and PCR, respectively. In addition, the soluble levels of MIF and TNFα in both studied groups were quantified by ELISA and MILLIPLEX assay, respectively.

**Results:**

The most frequent allele found in BC was the G (74.3%) and 6 (54%) in the variants ‐173G > C and ‐794 CATT_5‐8_, respectively, without significant differences in both groups. Nevertheless, the women with BC carriers ‐173*C and ‐794CATT_7_ have higher levels of MIF in comparison with CS. An increase of MIF (BC: 11.1 ng/mL vs CS: 5.2 ng/mL, *P* < .001) and TNFα (BC: 24.9 ng/mL vs CS: 9.9 pg/mL, *P < *.001) was found.

**Conclusion:**

The functional variants of *MIF* are not genetic susceptibility markers for BC. Nevertheless, the alleles ‐173*C and ‐794CATT_7_ are associated with the increase of MIF circulating in women with BC.

## 1 INTRODUCTION

Breast cancer (BC) is the leading cause of death in women and is defined as a heterogeneous disease of multifactorial origin,^1^, ^2^ according to data from the World Health Organization (WHO). There is a classification of BC defined by biomarkers by immunohistochemistry as estrogen receptor (ER), progesterone receptor (PR), receptor 2 of human epidermal growth factor (HER2), and Ki67 have been very useful to define molecular profiles,^3^, ^4^ which represent a guide for the diagnosis and treatment of patients.^4^ The molecular subtypes are classified in Luminal A, Luminal B, HER2, and triple negative (TN).^5^, ^6^ BC is a multifactorial disease in which pathogenesis merges several factors like environmental, genetic, and immunologic, where the proinflammatory cytokines highlight.^7^, ^8^, ^9^

Migration inhibitory factor is a pleiotropic cytokine that is involved in tumor pathology,^10^ because participates promoting inflammation, angiogenesis, and metastasis,^11^ besides MIF can regulate innate and adaptive immunity, it constitutively released by many cells including macrophages, B and T cells, dendritic cells, granulocytes, endocrine, endothelial, epithelial cells,^12^, ^13^ and also by tumor cells.^14^ Furthermore, it has been observed that MIF has a positive feedback mechanism with the production of TNFα, in vitro.^15^ In this regard, there are reports that mention that MIF promotes the tumor microenvironment through inflammation with the production of inflammatory cytokines including IL‐1β, IL‐6, and TNFα.^16^

The *MIF* gene is encoded on chromosome 22 (22q11.2) in humans; it consists of 3 exons,^12^, ^17^, ^18^ and it is composed of 115 amino acids with a molecular weight of 12.5 kDa, and its active form in the conformation homotrimer.^13^, ^17^

There are existing reports that establish functional variants associated with the increased expression the *MIF* (‐173G > C and ‐794 CATT_5‐8_).^19^ The promoter sequence of *MIF* has several specific binding sites for different transcription factors that regulate its expression, which include SP1, CREB, NFkB, Pit‐1, AP‐1, ICBP90, and HIF‐1α.^20^, ^21^, ^22^, ^23^, ^24^ The first functional variant of *MIF* is a single nucleotide polymorphism at ‐173 (G > C), which creates a binding site for protein activator‐4 (AP4) that increases promoter activity of *MIF*.^21^ The second site consists of the CATT repeats at ‐794 which repeats 5‐8 times, creating a binding site for the transcriptional factor Pit‐1, as well as ICBP90, which regulates the activity on this site.^24^ Furthermore, two studies suggest the association between *MIF* polymorphism ‐173 G > C with the risk of developing BC in the Chinese population.^18^, ^25^

MIF protein is related to the development and progression of the tumor through the production of proangiogenic factors such as VEGF.^14^ Besides, MIF when forming the complex with CD74 and CD44 (also, interacts with CXCR2, CXCR4, and CXCR7 chemokine receptors)^26^ activates different signaling pathways like ERK1/2, MAPK, PI3K/AKT, and NFkB, involved in proliferation, differentiation, survival, and inflammation.^17^, ^26^ Likewise, the increased soluble levels of MIF have been associated in patients with prostatic, colon, lung, and skin cancer, as well as BC.^27^

However, it is still unknown if this functional variant of the *MIF* gene and the soluble levels of MIF and TNFα are associated with BC in the western Mexican population. This study aimed to associate the functional variants of the *MIF* gene (‐173G > C and ‐94 CATT_5‐8_) with the soluble levels of MIF and TNFα in women with BC.

## 2 MATERIALS AND METHODS

For this cross‐sectional study, 152 women were diagnosed histopathologically with BC belonging to the "*Ella*" Binational Breast Cancer Study and 182 women as control subjects (CS); all the subjects were recruited from Mexican‐mestizo population. All women with BC and CS participants received a written informed consent, approved by the ethics committee from the *Universidad de Guadalajara* (CI‐9708). The participation of CS in this study was voluntary, and both groups signed a written informed consent. In addition, this study was performed according to the ethical principles for experiments involving humans stated on the Declaration of Helsinki. The women with BC, under pharmacology treatment with corticoids or chemotherapy, were not included in the present study.

### 2.1 Identification of molecular subtypes

The intrinsic molecular‐like subtypes were identified by immunohistochemistry, according to the St. Gallen consensus 2013 recommendations (2015‐updated): Luminal A‐like, Luminal B‐like, HER‐2, and triple negative (TN).^28^

### 2.2 Genotyping of functional variants of MIF gene (SNP ‐173G > C and STR CATT_5‐8_)

The blood samples were obtained from patients and control by venipuncture. Genomic DNA was extracted from peripheral blood leukocytes using Miller's technique and stored at −20°C until analysis. For the identification of SNP ‐173 G > C (rs755622), *MIF* was achieved by conventional polymerase chain reaction and restriction fragment length polymorphism (PCR‐RFLP) in a Thermal Cycler (TECHNE TC‐ 312) using the following primers: 5´ACT AAG AAA GAC CCG AGG C 3´ (Forward) and 5´GGG GCA CGT TGG TGT TTA C 3´ (Reverse), reported by Radstake et al^29^ PCR conditions were for ‐173G > C: an initial denaturation at 95°C for 4 minutes followed by 30 cycles of 30 seconds at 95°C, 30 seconds at 60°C, and 30 seconds at 72°C with a final extension of 4 minutes at 72°C. Amplification products of 366 bp were obtained. Subsequently, the fragment was digested with *Alu I* restriction endonuclease (New England biolabs, USA) by 3‐hour incubation at 37°C. Finally, the digestion was resolved on a 6% polyacrylamide gel stained, with 2% AgNO_3_. With respect to polymorphism, STR CATT_5‐8_ (rs5844572) was using the following primers: 5´TTG CAC CTA TCA GAG ACC 3´ (Forward) and 5´TCC ACT AAT GGT AAA CTG G 3´ (Reverse). PCR conditions were for ‐794 CATT_5‐8_ an initial denaturation at 95°C for 4 minutes followed by 35 cycles of 30 seconds at 95°C, 30 seconds at 60°C, and 30 seconds at 72°C with a final extension of 4 minutes at 72°C. Amplification products were visualized after electrophoresis on an 7% polyacrylamide gel stained with 2% AgNO_3_. Fragments of 220, 216, 212, and 208 bp represented the ‐794_8_, ‐794_7_, ‐794_6_, and ‐794_5_ alleles, respectively.

### 2.3 Quantification of MIF and TNFα plasma levels

The plasma was collected following centrifugation and stored immediately at −80°C until analysis. MIF soluble levels were measured by commercial ELISA kits (BioLegend® Legend MaxTM, Human active MIF, Cat. No. 438408). The MIF assay limit detection was 8 pg/mL. The optical density was immediately determined, using a microplate reader set to 450 and 570 nm, and TNFα was measured in a multiplex assay (MILLIPLEX®MAP Cat. No. HCYTOMAG‐60K) according to the manufacturer´s instructions.

### 2.4 Statistical analysis

The statistical analysis was performed using STATA software (v9.2) and GraphPad Prism (v5.0) software. Differences in characteristics between groups were analyzed with chi‐square test, Student's t test (parametric variables), and Mann‐Whitney *U* test and Kruskal‐Wallis test (nonparametric variables) (data presented as median and 25th to 75th percentiles) were used. The Hardy‐Weinberg equilibrium test and genotype and allele frequencies were calculated by chi‐square test or Fisher's exact test, when it was necessary. Odds ratios (OR) and 95% confidence intervals (95% CI). In this study, the sample size was calculated according to the minor allele frequency of the ‐173 G > C and ‐794 CATT_5‐8_ *MIF* gene polymorphism, using the Kelsey formula. A *P* value < .05 was considered to be statistically significant.

## 3 RESULTS

The clinical and demographic characteristics of BC are summarized in Table 1. The women with BC had a mean of 55 years and post‐menopausal state (70.6%), and also, around 83% of the women with BC were classified in clinical stage II‐IV, in agreement with the distribution present in Mexico. According to the classification with molecular subtypes, the Luminal A and Luminal B are the most predominant in this study with 70.2% of the cases, while HER2 and TN presented the 16.7% and 13.2%, respectively. In addition, the body mass index (BMI) of the majority of women with BC was overweight and obese with 37.3% and 33.3%, respectively, and also presented high Ki67 (>14%).

**Table 1:** Clinical and demographic characteristics in CS and women with BC

Variable	CS (n)	Percentage (%)	BC (n)	Percentage (%)
Mean age at diagnosis (y)				
Mean ± SD	40.6 ± 8		55.5 ± 13	
Hormone status				
Pre‐menopausal			31	18.2
Post‐menopausal			120	70.6
BMI				
< of 25	69	45.7	44	29.3
25 to 29.9	54	35.8	56	37.3
>30	28	18.5	50	33.3
Ki‐67				
Low			37	27.6
High			72	53.7
Indeterminate			25	18.7
TNM clinical stage				
I			24	16.4
II			74	50.7
III			40	27.4
IV			8	5.5
Molecular subtype				
Luminal A			41	28.5
Luminal B			60	41.7
HER2			19	13.2
TN			24	16.7

Note

Data provided in average, percentage, and n*.*

Abbreviations: CS, control subjects; BC, breast cancer.

### 3.1 The soluble levels of MIF and TNFα in women with BC and CS

**Figure 1:**
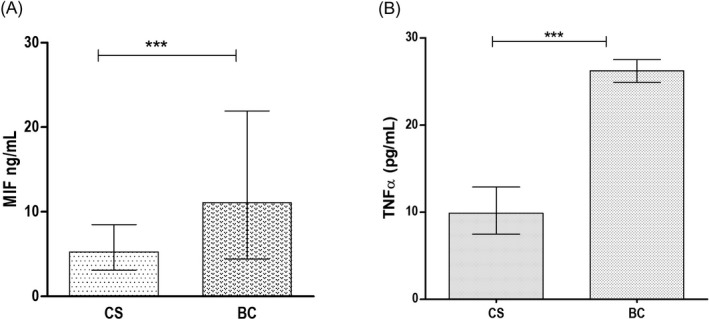
The soluble levels of MIF and TNFα in CS and BC. Comparison of MIF levels in CS vs BC (A). Comparison of TNFα levels in CS vs BC (B). CS, control subjects; BC, breast cancer. Data provided in median (p25‐p75). For the comparison, Mann‐Whitney *U* test was used (P** .01, P*** .001)

The soluble levels of MIF were increased in BC (11.1 ng/mL) compared to CS (5.2 ng/mL) [*P < *.001]. Likewise, the soluble levels of TNFα showed increased levels in women with BC (24.9 pg/mL) in comparison with CS (9.9 pg/mL) [*P < *.001] (Figure 1). In addition, we have tested the correlation between the soluble levels of MIF and TNFα; however, significant differences were not observed (*r* = −.069,* P* = .56). The soluble levels of MIF and TNFα stratified according to the molecular subtype (Luminal A, Luminal B, HER2, and TN) did not show significant differences between the groups (data not shown).

### 3.2 *MIF* gene ‐173 G > C polymorphism in the women with BC and CS

The functional variant analyzed was in Hardy‐Weinberg equilibrium in the CS (*P* = .94). The distribution of ‐173 G > C *MIF* polymorphism in women with BC and CS is shown in Table 2. We found that when comparing the genotypes of women with BC and CS, the significant differences were not shown. We observed a higher frequency of the G/G homozygous genotypes, followed by G/C heterozygote genotype.

**Table 2:** Genotype and allele frequencies of ‐173 G > C *MIF* polymorphism in CS and BC

Polymorphism ‐173 G > C	CS n = 182 (%)	BC n = 152 (%)	OR	CI 95%	P* value
Genotype					
G/G^a^	98 (53.9)	77 (50.6)	1		
G/C	75 (41.2)	72 (47.4)	1.22	0.76‐1.94	.37
C/C	9 (4.9)	3 (2)	0.42	0.07‐1.78	.19
Genetic model					
Dominant					
G/G	98 (53.9)	77 (50.6)	1		
G/C + C/C	84 (46.1)	75 (49.4)	1.13	0.72‐1.78	.56
Overdominant					
G/C	75 (41.2)	72 (47.4)	1.28	0.81‐2.02	.25
G/G + C/C	107 (58.8)	80 (52.6)	1		
Recessive					
C/C	9 (4.94)	3 (2)	0.38	0.06‐1.59	.14
G/G + G/C	173 (95.0)	149 (98)	1		
Allele					
G^a^	271 (74.5)	226 (74.3)	1		
C	93 (25.5)	78 (25.7)	1.0	0.69‐1.44	.97

Abbreviations: BC, breast cancer; CS, control subjects; OR, odds ratio; CI, confidence interval; P* value = X^2^ test

a Reference category.

We did not find any statistical association with ‐173 G > C *MIF* gene polymorphism in BC. Also, by applying a genetic model of dominant inheritance, the significant association between *MIF* ‐173 G > C polymorphism and the risk of BC for Mexican population (G/G vs GC + CC: OR = 1.13, 95% CI = 0.72‐1.78 *P* = .56) has not found. In addition, we found that the most frequent allele for women with BC was G (74.3%), followed by C (25.7%) (Table 2).

**Figure 2:**
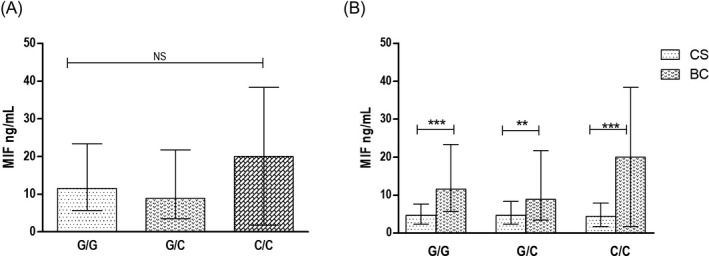
The soluble levels of MIF in ‐173 G > C *MIF* genotypes. Soluble levels of MIF according to genotypes in women with BC (A). Levels of MIF in CS vs BC by genotypes (B). CS, control subjects; BC, breast cancer. Data provided in median (p25‐75). For the comparison, Mann‐Whitney *U* test was used (P*** .001)

On the other hand, to determine whether this polymorphism is associated with soluble levels in women with BC, we compared the soluble levels of MIF and TNFα in women with BC according to ‐173G > C *MIF*. Nevertheless, significant differences were not observed, as well as by applying the dominant model. But, a tendency to the increase of soluble levels of MIF in C/C genotype (20.0 ng/mL) (Figure 2A) was observed, rather than G/C and G/G genotypes. Also, to compare the levels of MIF stratified by ‐173G > C *MIF* genotypes between women with BC and CS, we found that MIF is increased for each genotype in BC, while in CS shown similar levels in all the genotypes (Figure 2B). The soluble increased levels were observed in the same way in TNFα in women with BC compared with CS [*P < *.001] (data not shown).

When the genotypes of *MIF* were evaluated according to the molecular subtypes, no significant differences were observed. However, the HER2 molecular subtype of the carriers of the C/C genotype [20 ng/mL] has increased levels of MIF, unlike Luminal B [11.8 ng/mL] and TN [16.7 ng/mL] that showed higher levels in the homozygous G/G carriers (data not shown). Likewise, no significant differences were observed when comparing the soluble levels of TNFα according to the molecular subtype (data not shown).

#### 3.2.1 *MIF* gene ‐794 CATT_5‐8_ polymorphism in the women with BC and CS

With respect to the functional variant ‐794 CATT_5‐8_, the Hardy‐Weinberg equilibrium in the CS was also performed (*P* = .99). The distribution of ‐794CATT_5‐8_ *MIF* polymorphism in women with BC and CS is shown in Table 3. We found no significant differences when comparing the genotypes of women with BC and CS. We observed a higher frequency of the 5,6 heterozygote genotype, followed by 6,6 homozygous genotypes. However, no significant differences in the comparison of genotype frequency were found. Likewise, applying a genetic model of dominant inheritance significant differences was not observed in the genotype that contains the ‐794CATT_7_ allele [‐, ‐^a^ (‐,7 + 7,7)]. Furthermore, we found that the most frequent allele for women with BC was 6 (54%), followed by 7 (24%) (Table 3). We compared the genotypic and allelic frequencies of ‐794 CATT_5‐8_ MIF polymorphisms; however, we did not find significant differences.

**Table 3:** Genotype and allele frequencies of −794 CATT_5‐8_ *MIF* polymorphism in CS and BC

Polymorphism‐794 CATT_5‐8_	CS n = 182 (%)	BC n = 114 (%)	OR	CI 95%	P* value
Genotype					
5,5	3 (1)	7 (6)	4.1	0.84‐26.04	.04
5,6	55 (32)	37 (32)	1.18	0.60‐2.30	.59
5,7	12 (7)	0	–	–	–
6,6^a^	51 (28)	29 (25)	1		
6,7	49 (25)	27 (24)	0.96	0.47‐1.96	.92
7,7	12 (7)	14 (12)	2.0	0.76‐5.54	.11
Allele					
5	73 (21)	51 (22)	1.17	0.75‐1.83	.44
6^a^	206 (57)	122 (54)	1		
7	85 (22)	55 (24)	1.09	0.71‐1.67	.67
Genetic model (Do)					
‐, ‐a	109 (62)	73 (64)	1		
‐, 7 + 7,7	73 (38)	41 (36)	0.83	0.50‐1.39	.47

Abbreviations: Do, dominant inheritance genetic model; ‐,‐, Genotypes without the risk allele; BC, breast cancer; CS, control subjects; OR, odds ratio; CI, confidence interval; P* value = X^2^ test.

a Reference category.

**Figure 3:**
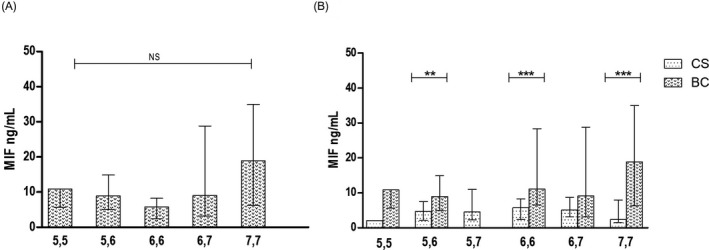
The soluble levels of MIF in ‐794 CATT_5‐8_ *MIF* genotypes in BC and CS. Soluble levels of MIF according to the genotypes in BC (A), and levels of MIF in CS vs BC according to genotypes (B). Data provided in median (p25‐p75). For the comparison, Mann‐Whitney *U* test was used (P** .01, P*** .001)

Regarding to the association in this polymorphism with the soluble levels of MIF and TNFα in women with BC significant differences by genotype were not shown, but we observed higher MIF levels in homozygous 7,7 carriers (18.8 ng/mL) in women with BC (Figure 3A). Nevertheless, we observed significant differences in the increased soluble levels of MIF in women with BC in comparison with CS in the heterozygous 5,6 carriers [8.95 ng/mL (BC) vs 4.6 ng/mL (CS) *P < *.01], homozygous 6,6 carriers [11.08 ng/mL (BC) vs 5.7 ng/mL (CS), *P < *.001)], and homozygous 7,7 carriers [18.8 ng/mL (BC) vs 2.3 ng/mL (CS), *P < *.001)] (Figure 3B).

On the other hand, when we compared the soluble levels of MIF in women with BC vs CS, carrying of the ‐794 CATT_7_ allele (‐,7 + 7,7), a higher levels of MIF were observed in BC (*P < *.05) [data not shown].

These results show that women with BC with allele 7 have higher soluble levels of MIF, which suggest that the allele ‐794 CATT_7_ similarly to 173*C could increase the MIF circulating in BC.

Subsequently, we observed that the soluble levels of TNFα were higher in the women with BC in comparison with CS (*P < *.001). Likewise, no significant differences were observed with the soluble levels of TNFα according to genotypes (data not shown). Furthermore, the soluble levels of MIF and TNFα were analyzed according to the molecular subtype; however, significant differences were not observed (data not shown).

## 4 DISCUSSION

In the present study, we have investigated the association of the functional variants of the *MIF* gene (‐173G > C and ‐794 CATT_5‐8_) with the soluble levels of MIF and TNFα in women with BC of western Mexico. BC is a major lethal cancer in female.^30^ Several studies have reported that presence of genetic factors as mutations and polymorphisms (*BRCA1/2*, *TP53*, *PTEN* and *MIF*) plays an important role in the development of BC, as well as proinflammatory cytokines, like MIF.^25^, ^31^ The human *MIF* gene is characterized by the presence of a single nucleotide polymorphism (‐173 G > C) and a microsatellite repeat (‐794 CATT) both in the promoter region.^11^ The involvement of *MIF* alleles in the development of cancer was first associated and described in prostate cancer.^19^

As far as we were concerned, this is the first study that evaluates the allelic and genotypic frequencies of ‐173 G > C and ‐794 CATT_5‐8_ polymorphisms *MIF* in women with BC from western Mexico.

Previously, only one study evaluates the ‐173 G > C polymorphism in women with BC with a frequency of 73.6% for the G allele and 23.7% for the C allele in Chinese population.^25^ This result is consistent with our report, because we found similar frequencies (‐173*G = 74.3% and ‐173*C = 25.7%).

However, in study of Lin et al, they associated the ‐173*C *MIF* polymorphisms with the risk of BC, demonstrating that ‐173*C allele increases the risk of BC especially in older women of Chinese population.^25^ Also, other study suggests that MIF ‐173G > C polymorphism was associated with the participation of developing cancer, preponderantly in prostate cancer.^23^

For the frequencies of ‐794 CATT_5‐8_, *MIF* polymorphism was similar to other studies that evaluated autoimmune diseases in patients from western Mexico,^32^ but there is no existing report that determines the distribution of this polymorphism in cancer from this population.

In a study of Wu et al, evaluating the ‐794 CATT_5‐8_ *MIF* polymorphism in early stage cervical cancer (ESCC), they found that ‐794 CATT_7_ allele is associated with ESCC.^27^

Nonetheless, in our results we did not find association between the functional ‐173*C and ‐794 CATT_7_ *MIF* gene and women with BC in from western Mexico. These differences could be attributed to the criteria for inclusion and the sample size, and they can be influenced by the racial differences between populations.^32^, ^33^ It is known that genetic factors play an important role in defining the host immune response, and since the genetics of Mexican population is the result of interbreeding of American native, European, Asian, and African subjects,^34^ this intensive mix could explain the observed differences. The inclusion of the geographical localization was substantial for this study, considering that the genetic variants may not have the same association with disease in different populations; for example, ‐173G > C has been related to BC in Chinese population.^33^ Similarly, the inclusion of studies from other geographic localization that have explored whether the MIF ‐173 G > C is associated with an increased risk cancer obtained the heterogeneous results. These results indicate the importance of geographic localization and ethnic origin in even in the same country.

Likewise, we have tested the potential relationship between the functional variants of *MIF* gene with the soluble levels of MIF and TNFα in women with BC and CS. In our study, the women with BC showed increased circulating levels of MIF and TNFα in comparison with CS consistent with other reports.^17^, ^35^ With respect to ‐173G > C polymorphism in our results, we observed higher soluble levels of MIF in women with BC carriers of C/C genotype, similar to women carriers of 7,7 genotype. In addition, when grouping the genotypes that have the allele ‐794 CATT_7_ (‐,7 + 7,7), a significant difference when compared with CS was observed (*P < *.05).

These results suggest that women carriers with the alleles −173*C and −794CATT_7_ have a tendency to increase circulating levels of MIF protein in this pathology, while the soluble levels of MIF in CS are similar in all the genotypes for both polymorphisms. A possible explanation for this observation could be that MIF has a synergistic effect with the other proinflammatory cytokines such as IL‐17A which are increased in women with BC unlike to CS.^36^ In this way, it has been observed that proinflammatory cytokines can induce the release of MIF.^7^

On the other hand, it has been reported that the presence of the C allele creates a binding site for the AP‐4 transcriptional factor and its presence is associated with greater expression of the gene,^37^ similarly to the ‐794 CATT_7_ which generates a binding site for the transcriptional factor Pit‐1, with the consequently increased expression of *MIF* gene.^19^ In addition, another transcriptional factor involved in the regulation of *MIF* gene was ICBP90, a protein that interacts directly with *MIF* ‐794 CATT_5‐8_ and that favors transcription of the *MIF*.^24^

Additionally, the functional variants of *MIF* are associated with overexpression of MIF that participates in tumor growth promoting angiogenesis and metastasis;^10^, ^26^ moreover, MIF can induce secretion of bFGF, VEGF, and IL‐8 when activates the MAPK, PI3K signaling pathways, which lead to the increased secretion of these proangiogenic factors that are necessary for blood vessel formation. Also, under hypoxic conditions, that is a characteristic of the tumor, HIF‐1α is expressed and induces the production of MIF playing a role in tumor angiogenesis.^11^ In addition, HIF‐1α downregulates the expression of e‐cadherin, responsible for the formation of focal adhesion complex, which favors the metastasis. Similarly, MIF induces the expression of matrix metalloproteinase which degrade the basal membrane which enhances the metastatic process.^11^

Furthermore, our results confirm previous reports that establish that TNFα is increased in women with BC.^16^, ^35^, ^38^

The high BMI could be a risk factor that contributes to inflammatory conditions as previously was reported in BC.^25^ Some reports establish that overweight and obesity are states of chronic inflammation, which contributes to the promotion of the production of inflammatory cytokines that maintain an inflammatory tumor microenvironment.^16^, ^35^, ^36^, ^38^ Nevertheless, when we compare the soluble levels of MIF and TNFα, significant differences were not found between CS and BC stratified according to the BMI (data not shown), because in our BC study group, other conditions could influence the soluble levels of MIF and TNFα, such as hormonal status and clinical stage or severity of disease.

We have tested the possible association of MIF and TNFα, but we have not found significant correlation. A possible explanation for this could be the lack of association of MIF and TNFα which play a role in the site of tumor where the inflammatory process is exacerbated; therefore, more studies are necessary for the determination of these cytokines in situ*.*

MIF is considered a pleiotropic cytokine that participates in innate and adaptive immunity regulating the inflammation, also considered as the link between chronic inflammation and cancer.^26^ However, the association of MIF with the development of BC is not conclusive.

To our knowledge, the results presented in this study provide the first findings conducted in women with BC from western Mexico with the functional variants of the *MIF* gene, as well as with the soluble levels of MIF and TNFα. These studies are important because recently different studies showed the evidence that the cytokines are generated by immune and tumor cells and are associated with the prognosis in patients with BC.

## 5 CONCLUSIONS

The functional variants of the *MIF* gene (‐173 G > C and ‐794 CATT_5‐8_) did not shown to be genetic susceptibility markers in BC in women of western Mexico population. Furthermore, the soluble levels of MIF and TNFα in women with BC were increased in comparison with CS, while the presence of the alleles ‐173*C and ‐794CATT_7_ shown a tendency of increase of circulating levels of MIF in women with BC.

## ETHICAL APPROVAL

This study was conducted conforming to the Declaration of Helsinki, and the research was approved by the ethical investigation, committee from each of the hospitals, and Universidad de Guadalajara (CI‐9708).

## CONSENT TO PARTICIPATE

Informed consent was obtained from each of the participant before being enrolled in this study.
